# Nationwide insights on early childhood neurodevelopment during a global health crisis: evidence from COVID-19 in South Korea

**DOI:** 10.7189/jogh.16.04026

**Published:** 2026-01-12

**Authors:** Ah-Young Kim, Hyunjoo Lee, Ji-Hoon Na, Hankil Lee, Young-Mock Lee

**Affiliations:** 1 Department of Pediatrics, Yonsei University College of Medicine, Gangnam Severance Hospital, Seoul, South Korea; 2 College of Pharmacy, Ewha Womans University, Seoul, South Korea

## Abstract

**Background:**

The COVID-19 pandemic has disrupted early childhood environments globally, raising concerns about its potential impacts on neurodevelopment. Although early childhood is a critical developmental period, large-scale evidence from South Korea – where strict social distancing and unique caregiving structures were in place – remains limited. We aim to evaluate age- and domain-specific neurodevelopmental outcomes among children aged 0–5 years before and during the pandemic, focusing on differences by age and sex.

**Methods:**

We analysed children aged 0–5 years using data from a national health screening programme and a pre–post comparison design with repeated cross-sectional data. We compared the pre-pandemic (July 2018–March 2020) and pandemic (April 2020–December 2021) periods. We categorised children into infants (9–12 months), toddlers (18–36 months), and preschoolers (42–71 months). We measured developmental outcomes using the Korean Developmental Screening Test across six domains: gross motor, fine motor, cognition, language, social skills, and self-help. We conducted multivariable logistic regression and difference-in-differences analyses.

**Results:**

We analysed 6 253 076 assessments from 2 797 459 children. Peer-level developmental status declined significantly during the pandemic across all age groups, with the most pronounced decrease among toddlers (adjusted odds ratio (aOR) = 0.92; 95% confidence interval (CI) = 0.91–0.92), followed by infants and preschoolers. The language domain experienced the greatest decline (aOR = 0.87; 95% CI = 0.86–0.88), whereas the gross motor domain showed significant improvement (aOR = 1.13; 95% CI = 1.11–1.15). Boys were more adversely affected than girls, particularly in gross motor and social skill domains.

**Conclusions:**

The COVID-19 pandemic led to significant developmental declines among young children, particularly in language and social domains and among toddlers. Boys were more adversely affected than girls, especially in language and socioemotional skills, highlighting sex-related vulnerabilities. Prioritising early screening and interventions targeting these key domains, alongside sex-sensitive strategies and caregiver support, will be essential to mitigate developmental disruptions during future pandemics.

Pandemics have a recurrent nature, and in this century alone, the world has experienced severe acute respiratory syndrome, Middle East respiratory syndrome, Ebola virus disease, and, most recently, the COVID-19 pandemic [1,2]. Each of these pandemics has had a profound impact on children, affecting them physically, mentally, and socially [3–5]. The COVID-19 pandemic has brought major changes in the environment necessary for children’s neurodevelopment.

The impact of the COVID-19 pandemic on early childhood neurodevelopment has been investigated in multiple countries, with findings highlighting heterogeneous effects across settings and age groups. In the USA, a study involving children aged <5 years reported delays in communication and problem-solving domains among the pandemic cohort, whereas fine and gross motor skills were largely unaffected [6]. In contrast, research conducted in China among infants aged <1 year identified delays in fine motor and communication skills [7]. In Japan, a longitudinal study that followed children aged one and three over a two-year period revealed significant delays in language concept development and in social interactions with adults among three-year-old children [8]. Collectively, these studies underscore that the developmental impact of the pandemic varies across countries and developmental stages.

Both age group and national context are critical considerations when evaluating the impact of the pandemic on child development. South Korea represents a unique setting, having implemented stringent social distancing policies during the pandemic. As an ultra-low-fertility country with a high proportion of only children, it relies heavily on early private education and grandparental caregiving within the context of an increasing number of dual-earner households. In addition, the country’s advanced healthcare system and comprehensive national data infrastructure enable population-level monitoring of child development across standardised screening intervals. This allows for precise assessment of age- and domain-specific neurodevelopmental changes during the pandemic and highlights the value of conducting comprehensive studies tailored to the Korean context.

In South Korea, a national health screening programme for infants and children is conducted at regular intervals, with seven assessments from birth to age five. The programme aims to systematically monitor growth and development and to identify developmental concerns at an early stage. Early childhood neurodevelopment encompasses five core domains, including motor, cognitive, language, socioemotional, and adaptive/self-care skills, which may be differentially affected by pandemic-related disruptions [9]. As part of the national screening programme, the Korean Developmental Screening Test for Infants and Children (K-DST) is used to assess six developmental domains: gross motor, fine motor, cognition (recognition), language, social skills, and self-help. The K-DST is administered in clinical settings through a parent-completed questionnaire comprising 40–60 age-specific items tailored to the child’s developmental stage. Developed to reflect the developmental characteristics of Korean children, the K-DST has demonstrated robust screening performance, with a reported sensitivity of 0.833 and specificity of 0.979 [10].

Therefore, we aimed to examine the broader impact of the COVID-19 pandemic on child development across different age groups in South Korea, with a focus on specific developmental domains. By identifying age-specific patterns of neurodevelopmental vulnerability, the findings may inform targeted intervention strategies and support the design of tailored educational policies to better prepare for future public health crises.

## METHODS

### Study design

In this pre-post comparison study, we used repeated cross-sectional data and analysed the nationwide K-DST data to examine changes in developmental status among children before and during the COVID-19 pandemic and to assess differential impacts by sex. We stratified developmental data for children aged 0–5 years into three age groups: infants, toddlers, and preschoolers. We categorised data collected between July 2018 and December 2021 into the pre-pandemic period (July 2018–March 2020) and the pandemic period (April 2020–December 2021). We adhered to the STROBE reporting guideline to ensure methodological rigour (Table S1 in the **Online Supplementary Document**) [11]. The requirement for informed consent was waived owing to the retrospective nature of the study, which complied with the ethical principles outlined in the Declaration of Helsinki.

### Data and study population

We obtained the K-DST data from the National Health Insurance Service (NHIS-2023-1-403). The data set included socioeconomic variables such as age, sex, insurance type, disability status, prematurity, vision and hearing screening results, and physical examination records, along with developmental assessment outcomes [12].

The study population comprised all children across South Korea who participated in the national health screening programme at designated intervals. We included 2 797 459 children who met the inclusion criteria. We classified participants who underwent health check-ups at 9–12 months as infants (age 0). Additionally, we categorised those who were examined at 18–24 months (one year old) and 30–36 months (two years old) as toddlers, and those who received check-ups at 42–48 months (three years old), 54–60 months (four years old), and 66–71 months (five years old) as preschoolers [13]. To ensure accurate comparisons, we only included children who received K-DST assessments within the specified periods and excluded those without corresponding data.

### Measures and variables

We assessed the impact of the pandemic using developmental outcomes from the K-DST. We categorised raw K-DST scores into three levels based on standard deviations from the mean: peer-level, requiring follow-up (<–1 SD), and advised for further assessment (<–2 SD) [14]. The primary independent variable was the period of assessment, divided into pre-pandemic and pandemic periods, with 1 April 2020 designated as the cut-off date to account for potential policy implementation lag effects [15]. We determined prematurity status based on health screening results obtained before one year of age. We defined disability status as having received an official disability grade designation from national authorities. Furthermore, we classified the insurance type as National Health Insurance or Medical Aid based on income level. National Health Insurance is the universal public health insurance system covering all citizens, whereas Medical Aid is a social security programme for economically disadvantaged populations.

### Statistical analysis

To examine the impact of the COVID-19 pandemic, we performed χ^2^ tests and multivariable logistic regression analyses to compare the pre-pandemic and pandemic periods. Each K-DST assessment served as the unit of analysis. In all models, we adjusted for potential confounders, including prematurity, disability status, sex, and insurance type. We accounted for repeated assessment measures within individuals using generalised estimating equations with an exchangeable correlation structure. Because we examined multiple developmental domains, we applied Bonferroni correction to adjust for multiple testing and control the family-wise error rate. We assessed multicollinearity using variance inflation factors and found no evidence of problematic multicollinearity. We evaluated model fit using Hosmer–Lemeshow goodness-of-fit tests, and calculated adjusted odds ratios (aORs) with 95% confidence intervals (CIs) to quantify the changes. We used these analyses to estimate overall differences in developmental outcomes between the pre-pandemic and pandemic periods across the entire population.

To further examine potential subgroup-specific effects, we conducted a difference-in-differences (DID) analysis focusing on sex differences. We employed the DID approach to assess whether the pandemic's impact on developmental outcomes differed between boys and girls. The DID analysis is a quasi-experimental design used to estimate the effect of an intervention by comparing changes over time between groups [16]. This approach relies on the parallel-trend assumption, which posits that in the absence of the pandemic, the developmental trajectories of boys and girls would have followed similar trends. Using females as the reference group, we estimated the effect of the pandemic on male K-DST outcomes through a logit model with the following specification: M_it_ = β_0_ + β_1_ × Sex + β_2_ × Time + β_3_ × Sex × Time +β_4_ × Prematurity + β_5_ × Disability + β_6_ × Insurance + ε_it_

where Mit represents the logit of the binary dependent variable for age group τ(0 = peer level; 1 = requiring follow-up or further assessment). The sex × time interaction term served as the DID estimator. We entered all relevant confounders as binary covariates.

We used SAS, version 9.4 (SAS Institute, Cary, NC, USA) for all analyses. We set the statistical significance at a two-sided *P* < 0.05.

## RESULTS

### Patient demographics

We analysed 6 253 076 health check-ups from 2 797 459 children, comprising 3 049 921 check-ups conducted in the pre-pandemic period and 3 203 155 during the pandemic period (**Table 1**). Of the total check-up records, 51.3% were boys and 48.7% were girls, with 14.5% classified as infants, 33.6% as toddlers, and 51.9% as preschoolers. There were no significant sex differences over time, whereas age distribution, insurance type, income level, preterm birth rate, and disability rate showed notable variation. A higher proportion of infants was examined before the pandemic, whereas a greater proportion of preschoolers underwent check-ups during the pandemic. In addition, preterm birth rates increased, while the proportion of children with disabilities decreased during the pandemic. The proportion of children enrolled in the Medical Aid programme also declined slightly during the pandemic period.

**Table 1:** Demographic characteristics at each K-DST assessment by study periods

	Pre-pandemic*	Pandemic*	*P*-value
**Total n**	3 049 921(100)	3 203 155(100)	
**Sex**			0.8643
Boy	1 564 486 (51.3)	1 642 870 (51.3)	
Girl	1 485 435 (48.7)	1 560 285 (48.7)	
**Age group**			<0.001
Infant	477 421 (15.7)	432 336 (13.5)	
Toddler	1 076 974 (35.3)	1 022 999 (31.9)	
Preschooler	1 495 526 (49.0)	1 747 820 (54.6)	
**Insurance†**			<0.001
NHI	3 016 771 (98.9)	3 167 732 (98.9)	
MA	32 526 (1.1)	27 132 (0.9)	
**Income†**			<0.001
Top	1 583 708 (51.9)	157 8895 (49.3)	
Middle	904 000 (29.6)	1 020 105 (31.9)	
Bottom	417 209 (13.7)	440 465 (13.8)	
**Preterm†**			<0.001
Yes	151 183 (5.0)	179 353 (5.6)	
No	2 689 055 (88.2)	2 817 514 (88.0)	
**Disabled**			<0.001
Yes	13 372 (0.4)	8899 (0.3)	
No	3 036 549 (99.6)	3 194 256 (99.7)	

K-DST – Korean Developmental Screening Test, MA – medical aid, NHI – national health insurance

*Values are presented as n (%).

†Percentages may not sum to 100% for insurance, income, and preterm birth due to missing data.

### Overall K-DST results

**Figure 1:**
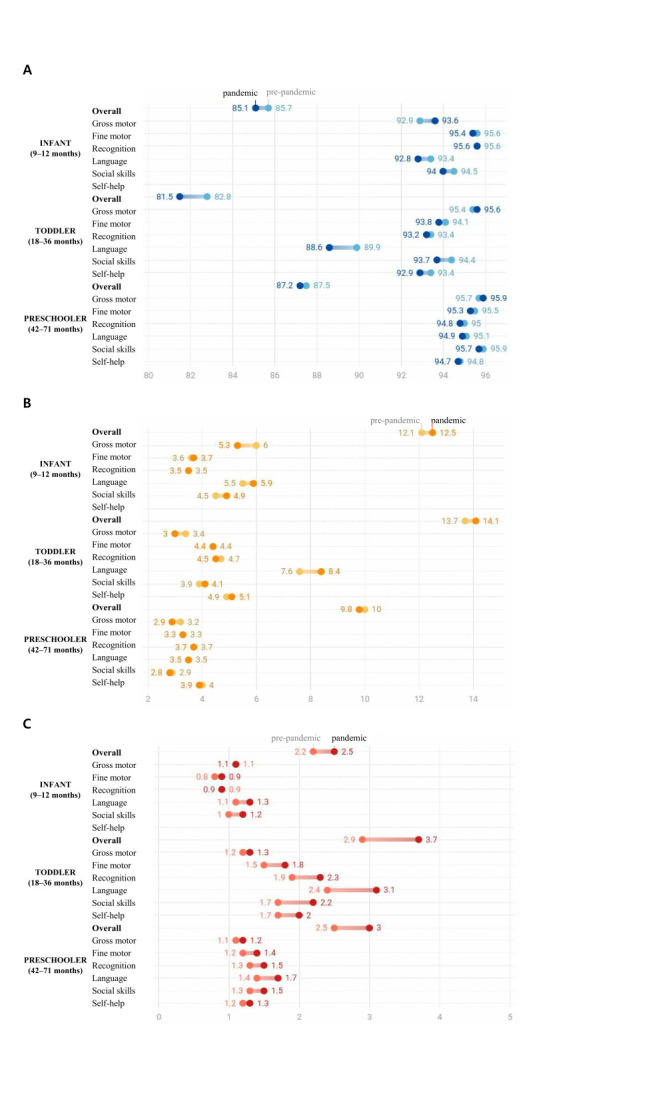
Distribution of Korean developmental screening test (K-DST) results by periods. **Panel A.** Peer level. **Panel B.** Follow-up requirement. **Panel C.** Referral for further assessment. K-DST – Korean Developmental Screening Test.

**Figure 2:**
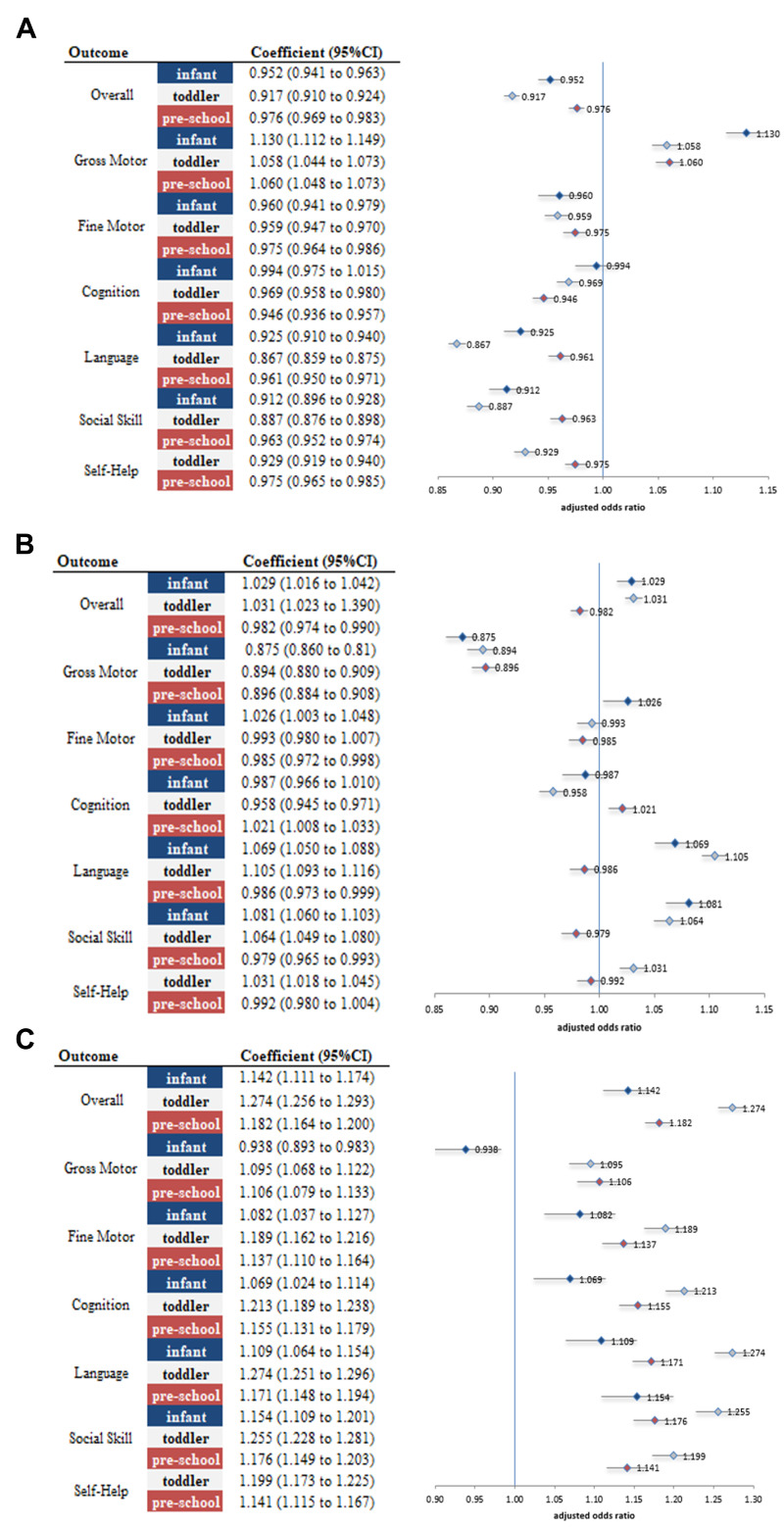
Adjusted odds ratios of K-DST outcomes by domain across age groups. **Panel A.** Peer level. **Panel B.** Follow-up requirement. **Panel C.** Referral for further assessment. *All models are adjusted for premature birth, disability status, insurance type, and sex.

Toddlers represented the most vulnerable group during the pandemic, exhibiting the greatest decline in overall developmental status compared with infants and preschoolers. Before the pandemic, the proportion of children at the peer level on the overall K-DST score was highest among preschoolers (87.5%), followed by infants (85.7%) and toddlers (82.8%) (**Figure 1**, Panel A; Table S2 in the **Online Supplementary Document**). During the pandemic, these proportions declined across all age groups, with the most pronounced decrease observed among toddlers (aOR = 0.917; 95% CI = 0.910–0.924), compared with infants (aOR = 0.952; 95% CI = 0.941–0.963) and preschoolers (aOR = 0.976; 95% CI = 0.969–0.983) (**Figure 2**, Panel A). The proportion of children requiring follow-up or further assessment also increased most notably among toddlers, further highlighting their heightened vulnerability during this period (**Figure 1**, Panels B and C; **Figure 2**, Panels B and C).

### Domain-specific K-DST results

In the gross motor domain, peer-level performance was highest before the pandemic (95.4–95.9%) and further improved during the pandemic (**Figure 1**, Panel A). The improvement was most pronounced among infants (aOR = 1.13; 95% CI = 1.11–1.15), although the odds of being advised for further assessment also increased among toddlers and preschoolers (**Figure 2**, Panel A). In the fine motor domain – one of the most developed domains prior to the pandemic (95.4–95.6%) – modest declines were observed across all age groups, particularly among infants (aOR = 0.96; 95% CI = 0.94–0.97) and toddlers (aOR = 0.96; 95% CI = 0.95–0.97).

The cognition domain was least affected, with little change observed among infants (aOR = 0.99; 95% CI = 0.95–1.02) and small but statistically significant declines among toddlers (aOR = 0.97; 95% CI = 0.96–0.98) and preschoolers (aOR = 0.95; 95% CI = 0.94–0.96).

The language domain experienced the most substantial decline. Peer-level performance decreased across all age groups, with the largest decline observed among toddlers (aOR = 0.87; 95% CI = 0.86–0.88), followed by infants and preschoolers.

Similarly, in the social skills domain, which was highly developed prior to the pandemic (95.7–95.9%), peer-level outcomes declined most notably among toddlers (aOR = 0.89; 95% CI = 0.88–0.90), followed by infants (aOR = 0.91; 95% CI = 0.90–0.93) and preschoolers (aOR = 0.96; 95% CI = 0.95–0.97).

Finally, in the self-help domain, declines were concentrated among toddlers (aOR = 0.93; 95% CI = 0.92–0.94) and preschoolers (aOR = 0.98; 95% CI = 0.97–0.99) (**Figure 2**, Panel A), accompanied by increases in the odds of requiring further assessment (**Figure 2**, Panel B).

### Comparison between sexes

The DID analysis revealed significant differences between boys and girls during the pandemic (**Table 2**). Overall, boys were more adversely affected than girls, particularly among infants and preschoolers. Among infants, boys exhibited a significantly greater decline in peer-level developmental status compared with girls, with a 3.1% larger reduction (DID = 0.97; 95% CI = 0.95–0.99). In contrast, no significant sex differences were observed among toddlers in overall K-DST outcomes, indicating that the pandemic’s impact was broadly similar between boys and girls in this age group.

**Table 2:** Impact of sex on K-DST outcomes: a DID analysis*†

	Overall K-DST	Gross motor	Fine motor	Cognition	Language	Social skills	Self-help
**Infant**							
Peer-level	0.969 (0.947–0.993)	1.048 (1.014–1.083)	1.005 (0.966–1.046)	0.959 (0.920–0.999)	0.971 (0.939–1.005)	0.961 (0.925–0.997)	
Follow-up requirement	1.022 (0.997–1.049)	0.945 (0.912–0.980)	0.995 (0.952–1.040)	1.039 (0.992–1.088)	1.023 (0.986–1.062)	1.050 (1.007–1.094)	
Referral for further assessment	1.050 (0.993–1.111)	0.999 (0.923–1.080)	0.974 (0.889–1.068)	1.042 (0.950–1.142)	1.043 (0.963–1.131)	0.991 (0.909–1.079)	
**Toddler**							
Peer-level	0.986 (0.971–1.001)	0.941 (0.915–0.967)	0.990 (0.965–1.015)	1.002 (0.978–1.026)	1.044 (1.024–1.065)	1.054 (1.027–1.083)	1.024 (0.999–1.049)
Follow-up requirement	0.992 (0.976–1.009)	1.056 (1.023–1.091)	1.008 (0.979–1.038)	0.977 (0.950–1.005)	0.942 (0.922–0.963)	0.930 (0.901–0.959)	0.968 (0.941–0.996)
Referral for further assessment	1.010 (0.979–1.043)	0.996 (0.943–1.053)	0.962 (0.914–1.012)	0.983 (0.940–1.028)	0.962 (0.925–1.001)	0.954 (0.910–1.000)	0.976 (0.929–1.025)
**Preschooler**							
Peer-level	0.963 (0.949–0.977)	0.896 (0.875–0.917)	0.980 (0.956–1.005)	0.995 (0.974–1.018)	0.995 (0.972–1.018)	0.959 (0.936–0.983)	0.990 (0.968–1.013)
Follow-up requirement	1.019 (1.003–1.035)	1.096 (1.067–1.126)	1.019 (0.989–1.049)	0.984 (0.959–1.009)	0.988 (0.962–1.015)	1.021 (0.992–1.051)	0.998 (0.973–1.024)
Referral for further assessment	1.044 (1.012–1.078)	1.069 (1.018–1.123)	1.017 (0.968–1.067)	1.013 (0.968–1.059)	1.002 (0.959–1.047)	1.026 (0.979–1.074)	1.019 (0.970–1.070)

DID – difference-in-differences, K-DST – Korean Developmental Screening Test

*By setting the change for females to 1, the magnitude of the change for males is represented.

†Values are presented as aOR (95% CI).

Among preschoolers, boys were more likely to be classified as requiring further assessment than girls (DID = 1.04; 95% CI = 1.02–1.07). This difference was especially notable in the gross motor domain, where boys had a 6.9% higher likelihood of needing further assessment than girls (DID = 1.07; 95% CI = 1.02–1.12).

## DISCUSSION

This study represents the first nationwide investigation to evaluate the impact of the COVID-19 pandemic on the neurodevelopment of children aged <5 years in South Korea, drawing on a uniquely large data set of approximately 6.3 million developmental assessment records. To examine this impact, we conducted a comprehensive analysis using national data and quasi-experimental methodologies. Our findings indicate that the pandemic had a significant adverse effect on overall developmental outcomes, with the most pronounced impact observed among toddlers. These results are consistent with global evidence [6,17–19], and the effects remained robust after adjusting for potential confounders, including preterm birth, disability status, and insurance type. The implementation of strict social distancing measures and widespread school closures likely contributed to these outcomes. Additionally, the pandemic period was characterised by heightened anxiety and stress among children [20], increased screen time [21], and reduced physical activity [22], all of which may have disrupted multiple developmental domains. Notably, children already at risk – such as those with disabilities or from lower socioeconomic backgrounds – likely experienced even greater deterioration due to restricted access to essential therapies, special education, and medical services [23,24]. Increased caregiving burdens and social isolation in these groups may have further exacerbated developmental challenges, underscoring the importance of targeted support for vulnerable populations during public health crises.

The impact of the pandemic differed substantially by age group, with toddlers showing the greatest developmental vulnerability, followed by infants and preschoolers. Toddlers exhibited the greatest vulnerability across all developmental domains except cognition. This heightened impact is likely attributable to the fact that this age represents a critical period of developmental milestones, during which interaction with the external environment is essential [25]. The social isolation imposed by the pandemic may have substantially disrupted their development [26]. In contrast, infants may have been less affected because their primary interactions were with family members, similar to pre-pandemic conditions. For preschoolers, the strong emphasis on education that begins at this stage in South Korea may have mitigated the adverse effects. One study found a notable increase in private education participation among preschoolers compared with two-year-olds, potentially buffering the impact of the pandemic on this age group [27]. These findings underscore the importance of age-specific interventions, including caregiver-directed developmental stimulation activities and education to ensure continuity of support even under pandemic conditions.

Although the overall impact of the COVID-19 pandemic on preschoolers was less pronounced than in other age groups, notable sex differences emerged. Boys exhibited a greater increase in developmental delays than girls, particularly in the gross motor and social skills domains across all age groups. Prior research suggests that boys typically engage in more physical activity and rely more heavily on gross motor play as they grow [28]. Such activities provide critical opportunities for social interaction, particularly through peer play. Consequently, restrictions on outdoor and group activities during the pandemic have disproportionately limited boys’ opportunities for both physical and social development, thereby exacerbating sex-based disparities in these domains. These findings underscore the need for sex-responsive strategies alongside age-specific interventions to address differential developmental impacts. Interventions that support boys’ motor and social skill development – such as indoor active play and structured role-play programmes (*e.g.* the Incredible Years Dinosaur Program) – may be particularly relevant in this context [29,30]

Language development emerged as the domain most severely affected by the COVID-19 pandemic, reflecting both environmental and demographic factors. Multiple factors – including mask-wearing and limited social interactions – likely contributed to disruptions in this domain. Masks obscure the mouth, making it difficult for children to observe lip movements and accurately perceive speech, both of which are critical for language acquisition [31]. Additionally, reduced opportunities for interaction in preschools and daycare centres decreased children’s daily exposure to language, potentially causing delays in language development. Pandemic-related restrictions on social interactions, particularly outside the home, further compounded these effects. Although children may have spent more time with their parents at home, elevated levels of household stress likely reduced the quality of parent-child interactions during this period [32]. In South Korea, where the proportion of only children is high, opportunities for daily language and social interactions with siblings are limited. This demographic characteristic may have further exacerbated the lack of language stimulation during the pandemic.

In contrast to other domains, gross motor development showed unexpected improvements during the pandemic. Prior research has indicated mainly that gross motor development was not substantially affected by the pandemic. For example, a USA study on over 50 000 children aged <5 years found no significant changes in gross or fine motor development during the pandemic [6], and studies in China and Ireland similarly reported no delays among infants aged 6–12 months [7,33]. In contrast, data from Uruguay linked reduced physical activity to declines in motor development [22]. While our findings differ from most prior studies, they align with results from a Korean study of children aged 30–36 months and a large-scale study in Colorado, both of which reported improvements in gross motor skills during the pandemic [34]. The observed improvements in gross motor development in South Korea are best explained by changes in the assessment context, particularly increased parental observation during the pandemic, rather than actual gains in ability. During this period, parents – including those in dual-earner households – spent more time with their children, which has been shown to enhance the accuracy and sensitivity of parental reporting, thereby contributing to higher observed scores in gross motor assessments [35]. Furthermore, the shift from typically sedentary activities in institutional settings to more active home-based routines is consistent with observed changes in physical activity levels during home confinement reported in previous studies [36,37], which likely contributed to the trend. These findings suggest that during pandemic periods, educational efforts may be more effectively directed toward language, cognitive, and social domains, rather than gross motor development.

This study has several limitations. First, during the COVID-19 pandemic, participation rates in well-child check-ups decreased as many parents were reluctant to visit healthcare facilities, leading to delays in developmental assessments. To address this issue, we included only children who underwent developmental screening at the appropriate age and excluded those assessed later. However, this exclusion may have introduced selection bias, depending on the omitted population's characteristics. Nevertheless, the overall sociodemographic characteristics of the included population remained broadly comparable across periods (**Table 1**). Second, the degree to which each child was affected by the pandemic likely varied; however, this heterogeneity could not be fully captured due to the limitations of claims data. While key variables such as daycare or preschool attendance, number of siblings, and prenatal exposure to severe acute respiratory syndrome-CoV-2 were unavailable, we adjusted for several important factors, including prematurity, disability, and socioeconomic status, to account for potential confounding. Third, because the K-DST is a parent-reported tool, differences in parental observation contexts may have influenced the results. Parental observation patterns may have changed during the pandemic as parents spent more time at home with their children. However, this limitation was partially mitigated by applying the same instrument across both periods and leveraging a large sample size, which likely reduced variability attributable to contextual differences. Finally, because the data were aggregated into two time periods without multiple pre-intervention intervals, we were unable to formally test the parallel pre-trend assumption underlying the DID analysis. This limitation should be considered when interpreting the DID estimates, as unobserved differences in pre-pandemic developmental trajectories between boys and girls cannot be entirely ruled out.

## CONCLUSIONS

The COVID-19 pandemic led to significant developmental declines among young children, particularly among toddlers and in language and social interaction domains, which are critical for subsequent cognitive and academic development. Boys experienced greater adverse impacts than girls, especially in language and socioemotional skills, indicating sex-related vulnerabilities. These findings call for targeted preparedness strategies to protect child development during future public health crises. Language and social development programmes should be prioritised, with tailored interventions for toddlers and boys through enhanced caregiver guidance and structured interaction programmes. In addition, stronger cross-sector collaboration between health, education, and social services is essential to support vulnerable groups, including children with disabilities and those from lower socioeconomic backgrounds. Further longitudinal and follow-up studies are needed to assess whether these developmental delays persist, recover, or widen over time, and to identify modifiable factors that can inform sustainable and targeted interventions.

## Additional material


Online Supplementary Document
