# Supplementary material for: Nationwide insights on early childhood neurodevelopment during a global health crisis: evidence from COVID-19 in South Korea
Source: J Glob Health. 2026 Jan 12;16:04026. doi: 10.7189/jogh.16.04026 (PMC12796865; doi:10.7189/jogh.16.04026)
Supplement: Online Supplementary Document [file jogh-16-04026-s001.pdf]

Supplement to: Kim AY, Lee H, Na JH, Lee H, Lee YM. Nationwide insights on early childhood neurodevelopment during a global health crisis: evidence from COVID-19 in South Korea. J Glob Health. 2026;16:04026.

Table S1. STROBE Statement—checklist of items that should be included in reports of observational studies

	Item No.	Recommendation	Page No.	Relevant text from manuscript
**Title and abstract**	1	(*a*) Indicate the study's design with a commonly used term in the title or the abstract	Abstract – Method	… a pre–post comparison design with repeated cross-sectional data.
		(*b*) Provide in the abstract an informative and balanced summary of what was done and what was found	Abstract – Conclusion	The COVID-19 pandemic led to significant developmental declines among young children, particularly among toddlers and in language and social interaction domains…
**Introduction**				
Background/rationale	2	Explain the scientific background and rationale for the investigation being reported	Background, paragraph 2	Collectively, these studies underscore that the developmental impact of the pandemic differs according to country and developmental stage.
Objectives	3	State specific objectives, including any prespecified hypotheses	Background, paragraph 4	This study aimed to examine the broader impact of the COVID-19 pandemic on child development across different age groups in South Korea, with a focus on specific developmental domains.
**Methods**				
Study design	4	Present key elements of study design early in the paper	Methods –	This pre–post comparison study

		Study Design	using repeated cross-sectional

				data analyzed the nationwide K-DST data…
Setting	5	Describe the setting, locations, and relevant dates, including periods of recruitment, exposure, follow-up, and data collection	Methods – Study Design	Data collected between July 2018 and December 2021 were categorized into the pre-pandemic period (July 2018–March 2020) and the pandemic period (April 2020–December 2021)
Participants	6	(*a*) *Cohort study*—Give the eligibility criteria, and the sources and methods of selection of participants. Describe methods of follow-up<br>*Case-control study*—Give the eligibility criteria, and the sources and methods of case ascertainment and control selection. Give the rationale for the choice of cases and controls<br>*Cross-sectional study*—Give the eligibility criteria, and the sources and methods of selection of participants	Methods – Data and Study Population	The study population comprised all children across South Korea who participated in the national health screening program… A total of 2,797,459 children who met the inclusion criteria were included in the analysis.
		(*b*) *Cohort study*—For matched studies, give matching criteria and number of exposed and unexposed<br>*Case-control study*—For matched studies, give matching criteria and the number of controls per case		Not applicable
Variables	7	Clearly define all outcomes, exposures, predictors, potential confounders, and effect modifiers. Give diagnostic criteria, if applicable	Methods – Measures and Variables	Raw K-DST scores were categorized into three levels based on standard deviations from the mean: peer-level, requiring follow-up (below –1 SD), and advised for further assessment (below –2 SD). The primary independent variable was the period of assessment, divided into pre-pandemic and pandemic periods.

Data sources/ measurement	8*	For each variable of interest, give sources of data and details of methods of assessment (measurement). Describe comparability of assessment methods if there is more than one group	Methods – Data and Study Population	K-DST data were obtained from the National Health Insurance Service (NHIS-2023-1-403). The dataset included socioeconomic variables such as age, sex, insurance type, disability status, prematurity, vision and hearing screening results, and physical examination records.
Bias	9	Describe any efforts to address potential sources of bias	Discussion – Limitations	To ensure accurate comparisons, only children who received K-DST assessments within the specified periods were included… This exclusion may have introduced selection bias… Nevertheless, the sociodemographic characteristics remained broadly comparable across periods.
Study size	10	Explain how the study size was arrived at	Methods – Data and Study Population	A total of 2,797,459 children who met the inclusion criteria were included in the analysis.

Continued on next page

Quantitative variables	11	Explain how quantitative variables were handled in the analyses. If applicable, describe which groupings were chosen and why	Methods – Measures and Variables	Raw K-DST scores were categorized into three levels based on standard deviations from the mean…
Statistical methods	12	(*a*) Describe all statistical methods, including those used to control for confounding	Methods – Statistical Analysis	Chi-square tests and multivariable logistic regression analyses were performed…
		(*b*) Describe any methods used to examine subgroups and interactions	Methods – Statistical Analysis	Potential confounders, including prematurity, disability status, sex, and insurance type, were adjusted for in all models…
		(*c*) Explain how missing data were addressed	Discussion-limitation	To address this issue, we included only children who underwent developmental screening at the appropriate age and excluded those assessed later
		(*d*) *Cohort study*—If applicable, explain how loss to follow-up was addressed<br>*Case-control study*—If applicable, explain how matching of cases and controls was addressed<br>*Cross-sectional study*—If applicable, describe analytical methods taking account of sampling strategy	Methods – Statistical Analysis	Bonferroni correction was applied to adjust for multiple testing and control the family-wise error rate.
		(*e*) Describe any sensitivity analyses		Not applicable – no sensitivity analyses were conducted in this study.
**Results**				
Participants	13*	(a) Report numbers of individuals at each stage of study—eg numbers potentially eligible, examined for eligibility, confirmed eligible, included in the study, completing follow-up, and analysed	Results – Patient Demographics	A total of 6,253,076 health check-ups from 2,797,459 children were analyzed, comprising 3,049,921 in the pre-pandemic period and 3,203,155 during the pandemic period.

		(b) Give reasons for non-participation at each stage		Not applicable – this study used routinely collected nationwide data; individual-level participation or attrition was not tracked.
		(c) Consider use of a flow diagram		Not applicable – as this was a repeated cross-sectional analysis of administrative data, participant flow was not relevant.
Descriptive data	14*	(a) Give characteristics of study participants (eg demographic, clinical, social) and information on exposures and potential confounders	Results – Patient Demographics	Of the total check-up records, 51.3% were boys and 48.7% were girls, with 14.5% classified as infants, 33.6% as toddlers, and 51.9% as preschoolers
		(b) Indicate number of participants with missing data for each variable of interest	Table 1	
		(c) *Cohort study*—Summarise follow-up time (eg, average and total amount)		Not applicable
Outcome data	15*	*Cohort study*—Report numbers of outcome events or summary measures over time		*Not applicable*
		Case-control study—Report numbers in each exposure category, or summary measures of exposure		*Not applicable*
		Cross-sectional study—Report numbers of outcome events or summary measures	Result-overall K-DST	"Peer-level developmental status declined significantly during the pandemic across all age groups, with the most pronounced decrease among toddlers (aOR, 0.92; 95% CI, 0.91–0.92), followed by infants and preschoolers. The language domain experienced the greatest decline (aOR, 0.87; 95% CI, 0.86–0.88), whereas the gross motor domain showed significant improvement (aOR, 1.13; 95% CI, 1.11–1.15).

Main results	16	(*a*) Give unadjusted estimates and, if applicable, confounder-adjusted estimates and their precision (eg, 95% confidence interval). Make clear which confounders were adjusted for and why they were included	Results / Discussion	Chi-square and logistic regression analyses revealed significant developmental declines among toddlers and in language and social domains… Boys experienced greater adverse impacts than girls
		(b) Report category boundaries when continuous variables were categorized	Methods – Measures and Variables	Raw K-DST scores were categorized into three levels based on standard deviations from the mean: peer-level, requiring follow-up (below –1 SD), and advised for further assessment (below –2 SD)
		(c) If relevant, consider translating estimates of relative risk into absolute risk for a meaningful time period		Not applicable – this study used adjusted odds ratios from cross-sectional comparisons rather than longitudinal risk estimates.

Continued on next page

Other analyses	17	Report other analyses done—eg analyses of subgroups and interactions, and sensitivity analyses	Result	Domain-specific analyses showed the greatest decline in language, followed by social skills, whereas gross motor improved during the pandemic
**Discussion**				
Key results	18	Summarise key results with reference to study objectives	Discussion – 1st paragraph	The COVID-19 pandemic led to significant developmental declines among young children, particularly among toddlers and in language and social interaction domains…
Limitations	19	Discuss limitations of the study, taking into account sources of potential bias or imprecision. Discuss both direction and magnitude of any potential bias	Discussion – Limitations	Participation rates in well-child check-ups decreased during the pandemic… we included only children assessed at the appropriate age… this exclusion may have introduced selection bias.
Interpretation	20	Give a cautious overall interpretation of results considering objectives, limitations, multiplicity of analyses, results from similar studies, and other relevant evidence	Discussion / Conclusion	Educational efforts may be more effectively directed toward language, cognitive, and social domains… These findings call for targeted preparedness strategies to protect child development during future public health crises
Generalisability	21	Discuss the generalisability (external validity) of the study results	Conclusion	Findings from this nationwide dataset of over 2.7 million children may inform future public health preparedness strategies in similar contexts.
**Other information**				

Funding	22	Give the source of funding and the role of the funders for the present study and, if applicable, for the original study on which the present article is based	Funding	This research was supported by the Basic Science Research Program through the National Research Foundation of Korea (NRF), funded by the Ministry of Education (No. RS-2023-00276504).

*Give information separately for cases and controls in case-control studies and, if applicable, for exposed and unexposed groups in cohort and cross-sectional studies.

Note: An Explanation and Elaboration article discusses each checklist item and gives methodological background and published examples of transparent reporting. The STROBE checklist is best used in conjunction with this article (freely available on the Web sites of PLoS Medicine at http://www.plosmedicine.org/, Annals of Internal Medicine at http://www.annals.org/, and Epidemiology at http://www.epidem.com/). Information on the STROBE Initiative is available at www.strobe-statement.org.

Table S2. Distribution of Korean developmental screening test (K-DST) results by periods

		**INFANT (9–12 months)**						**TODDLER (18–36 months)**						**PRESCHOOLER (42–71 months)**					
		Peer-level (%)		Require follow-up (%)		Advised for further assessment (%)		Peer-level (%)		Require follow-up (%)		Advised for further assessment (%)		Peer-level (%)		Require follow-up (%)		Advised for further assessment (%)	
		Pre-	pandemic	Pre-	pandemic	Pre-	pandemic	Pre-	pandemic	Pre-	pandemic	Pre-	pandemic	Pre-	pandemic	Pre-	pandemic	Pre-	pandemic
Overall	All	85.7	85.1	12.1	12.5	2.2	2.5	82.8	81.5	13.7	14.1	2.9	3.7	87.5	87.2	10.0	9.8	2.5	3.0
	Boy	83.8	82.8	13.7	14.2	2.5	3.0	78.2	76.6	17.0	17.4	4.7	6.0	85.0	84.5	11.6	11.5	3.3	3.0
	Girl	87.8	87.4	10.4	10.6	1.8	2.0	85.7	86.6	10.3	10.6	2.2	2.8	90.1	90.1	8.3	8.0	1.7	1.9
Gross motor	All	92.9	93.6	6.0	5.3	1.1	1.1	95.4	95.6	3.4	3.0	1.2	1.3	95.7	95.9	3.2	2.9	1.1	1.2
	Boy	92.5	93.%	6.3	5.4	1.2	1.2	95.0	95.1	3.5	3.2	1.5	1.7	95.5	95.5	3.1	3.0	1.4	1.5
	Girl	93.3	93.8	5.7	5.2	1.0	1.0	95.9	96.2	3.3	2.9	0.9	0.9	96.0	96.4	3.3	2.8	0.7	0.8
Fine motor	All	95.6	95.4	3.6	3.7	0.8	0.9	94.1	93.8	4.4	4.4	1.5	1.8	95.5	95.3	3.3	3.3	1.2	1.4
	Boy	95.2	95.0	3.8	3.9	1.0	1.1	92.4	92.0	5.5	5.5	2.1	2.5	93.6	93.4	4.7	4.7	1.7	2.0
	Girl	96.0	95.8	3.3	3.4	0.6	0.7	95.9	95.8	3.2	3.2	0.9	1.1	97.4	97.4	1.9	1.8	0.7	0.8
Recognition	All	95.6	95.6	3.5	3.5	0.9	0.9	93.4	93.2	4.7	4.5	1.9	2.3	95.0	94.8	3.7	3.7	1.3	1.5
	Boy	94.7	94.6	4.2	4.2	1.1	1.2	91.6	91.3	5.8	5.5	2.6	3.2	94.1	93.8	4.2	4.2	1.8	2.0
	Girl	96.6	96.6	2.8	2.7	0.6	0.7	95.3	95.2	3.5	3.4	1.1	1.4	96.0	95.8	3.2	3.3	0.8	0.9
Language	All	93.4	92.8	5.5	5.9	1.1	1.3	89.9	88.6	7.6	8.4	2.4	3.1	95.1	94.9	3.5	3.5	1.4	1.7
	Boy	91.7	90.9	6.9	7.4	1.4	1.7	86.6	84.9	10.0	10.8	3.4	4.3	93.6	93.4	4.4	4.3	2.0	2.3

	Girl	95.2	94.9	4.0	4.3	0.8	0.9	93.5	92.4	5.1	5.8	1.4	1.8	96.6	96.4	2.6	2.6	0.8	1.0
Social skills	All	94.5	94.0	4.5	4.9	1.0	1.2	94.4	93.7	3.9	4.1	1.7	2.2	95.9	95.7	2.9	2.8	1.3	1.5
	Boy	92.9	92.1	5.8	6.4	1.3	1.5	92.4	91.7	5.1	5.3	2.5	3.0	94.9	94.6	3.4	3.3	1.8	2.0
	Girl	96.2	96.0	3.1	3.3	0.7	0.8	96.4	95.9	2.6	2.9	1.0	1.3	96.9	96.9	2.3	2.2	0.8	0.9
Self-help	All	NA	NA	NA	NA	NA	NA	93.4	92.9	4.9	5.1	1.7	2.0	94.8	94.7	4.0	3.9	1.2	1.3
	Boy	NA	NA	NA	NA	NA	NA	90.8	90.2	6.8	6.9	2.4	2.9	93.2	93.0	5.2	5.1	1.6	1.9
	Girl	NA	NA	NA	NA	NA	NA	96.1	95.8	3.0	3.1	0.9	1.1	96.6	96.5	2.7	2.7	0.7	0.8
